# The misunderstanding of the R Classification—a survey amongst medical specialties treating breast cancer

**DOI:** 10.1007/s00428-024-03876-8

**Published:** 2024-07-22

**Authors:** Sandra Sunitsch, Philipp Fischer, Gudrun Pregartner, Peter Regitnig

**Affiliations:** 1 Diagnostic and Research Institute of Pathology, Medical University of Graz, Neue Stiftingtalstraße 6, 8010 Graz, Austria; 2 Institute for Medical Informatics, Statistics and Documentation, Medical University of Graz, Auenbruggerplatz 2, 8036 Graz, Austria

**Keywords:** Breast cancer, TNM Classification of Malignant Tumours, R Classification, Resection Margin, Tumour board

## Abstract

Frequent discussions in the tumour board about the Residual tumour (R) Classification of the UICC’s “TNM Classification of Malignant Tumours”, especially in the case of breast surgery specimens, raised the question about differing interpretations amongst different medical specialties. Thus, we designed a survey about the R Classification with a special focus on breast cancer specimens. An online survey was conducted, where a web link to the survey was distributed via email to various medical professional societies dealing with breast cancer in Austria and Germany with the request to distribute the link to their members. The study population consisted of physicians of all educational levels of different medical professions, who deal with breast carcinomas in their daily routine. Two hundred two participants, of which 160 (79.2%) have more than 10 years’ professional experience, took part in the survey; 88 (43.6%) were surgeons/gynaecologists, 80 (39.6%) pathologists, 19 (9.4%) radiation oncologists/ therapists, 8 (4.0%) radiologists, and 7 (3.5%) oncologists. We show that the R Classification is not completely mastered by anyone and that there are significant differences in the interpretation of the R Classification between different medical specialties. For better differentiation between the residual tumour (R Classification) of the TNM and a pure resection margin assessment, we suggest the use of a Resection margin (Rm) Classification to avoid further misunderstandings. To assist better multidisciplinary cooperation and to ensure better patient care all medical disciplines should be educated about the actual meaning and correct application of the R Classification.

**Supplementary Information:**

The online version contains supplementary material available at 10.1007/s00428-024-03876-8.

## Introduction

Breast cancer is the most prevalent cancer in women worldwide, accounting for 24% of all female cancers [1]. The recommended treatment for early-stage disease is a breast conserving surgery (BCS) [2]. In addition to the histopathological diagnosis including the “TNM Classification of Malignant Tumours” of the Union for International Cancer Control (UICC), the attending physicians expect a final Residual tumour (R) Classification by the pathologist. This classification describes the presence or absence of residual tumour after treatment, the effects of therapy, influences further therapeutic procedure, and is a strong prognostic predictor [3, 4]. But many physicians use “R” as a sole evaluation for the resection margin and consequently use “R0” synonymously for tumour-free resection margins. This applies not only to clinicians but also to pathologists. However, the residual tumour is not just a pure resection margin assessment [5]. To clarify problems on the subject of the R Classification, Wittekind et al. have written many articles and further explanations in the TNM Supplement [3, 4, 6–10]. Besides the R Classification, there are a number of suggestions dealing with the complex issue of the resection margin in breast carcinomas [11–15]. For example, in the common guidelines for invasive breast cancers with/without Ductal Carcinoma in situ (DCIS), “no ink on tumour” is recommended for either DCIS or invasive cancer cells. For a pure DCIS treated by BCS the optimal margin width should be at least 2 mm [15–24]. This complexity often leads to confusion between the R Classification and the resection margin assessment which results in animated discussions in the multidisciplinary tumour board.

Nevertheless, a uniform and correct application of the R Classification and the guidelines is essential. For this reason, we conducted the first large online survey on this topic within Germany and Austria, covering an area with a total population of 92 million people [25, 26], in order to highlight the inconsistencies between the different medical groups.

## Materials and methods

### Ethics statement

We performed an anonymous online survey as part of a diploma thesis [27] amongst physicians who participated voluntarily. No patient data was used. No benefit or disadvantage was associated with participation or non-participation. The identity of the participants was unknown and thus no informed consent was necessary.

The study was reviewed and approved by the local ethics committee of the Medical University of Graz (ID: 33–611 ex20/21).

### Creation and content of the survey

Guided by a literature review, we designed a survey about the R Classification with special focus on breast carcinomas. Altogether, the questionnaire consisted of 19 questions, including single and multiple-choice items.

The questionnaire was divided into three sections: “General” concerning professional expertise, the use of different guidelines and general questions concerning the R Classification, “General TNM Classification” and “Specific Questions Regarding Breast Cancer Diagnosis” with specific case examples. The individual sections contained three to nine questions. The questionnaire can be found in the supplement (S1).

### Implementation and distribution of the survey

The study population consisted of Austrian and German physicians of all educational levels from different medical professions, namely general surgeons, plastic surgeons and gynaecologists (hereafter referred to only as surgeons, since the medical disciplines mentioned are surgically active), oncologists, and radiologists, who deal with breast carcinomas in their daily routine.

The survey was conducted online using the software Lime Survey [28]. A web link to the survey was distributed via email to six Austrian and German medical societies dealing with breast cancer. The link was sent to these organisations with the request to distribute the survey to physicians participating in the treatment of breast cancer. The survey was available from the 23rd of June to the 22nd of October 2021. One email reminder was sent in order to increase the response rate four weeks before the end of the survey. No further actions were taken to contact study participants.

### Data analysis

All responders that completed/answered at least one survey question and indicated their medical specialty as well as their professional experience were considered for further analysis.

All survey items were analysed separately and summarized using absolute and relative frequencies. Group comparisons between the different medical specialties (five groups) as well as between different levels of experience (four groups) were performed using Fisher’s exact test. Post hoc tests were performed when the overall test was significant (*p* < 0.05). Bonferroni corrections were used for the interpretation of the post hoc tests, resulting in significance levels of 0.005 for medical specialty (10 pairwise comparisons).

For several items, it was possible to define an objective “correct” answer according to the guidelines. These were additionally analysed dichotomized into “correct” and “false”. For one question with multiple options (A4), each of which could have been selected and each of which was either deemed correct or false, a “sum score” was formed. The number of correct answers was then compared between the groups using the Kruskal–Wallis test and post hoc Mann–Whitney *U* tests.

All statistical analyses were conducted with R version 4.2.1.

## Results

### Demographic background

Of the 202 participants, 88 (43.6%) were surgeons, 80 (39.6%) pathologists, 19 (9.4%) radiation oncologists, 8 (4.0%) radiologists, and 7 (3.5%) oncologists.

Nineteen (9.4%) physicians with 0–5 years of experience, 23 (11.4%) with 6–10 years, 38 (18.8%) with 11–15 years, and 122 (60.4%) with more than 15 years of experience took part in the survey. Whereas 73/88 (83.0%) surgeons, 59/80 (73.8%) pathologists, and 13/19 (68.4%) radiation oncologists with an experience of more than 10 years answered the survey, the responses from specialties with low participation were exclusively from radiologists and oncologists with more than 10 years of experience.

### “General Section”

As shown in Table 1, there were significant differences regarding the use of guidelines and classifications between the medical specialties for the German AGO (Arbeitsgemeinschaft für Onkologie) S3 Guideline (*p* < 0.001), the St. Gallen International Consensus Guidelines (*p* = 0.003), the NCCN Clinical Practice Guidelines (*p* < 0.001), the ASTRO Consensus Guidelines (*p* < 0.001), the TNM Classification of the UICC (*p* < 0.001), and the AJCC Cancer Staging Manual (*p* < 0.001). Whereas all 19 (100%) radiation oncologists used the S3 Guideline, only 3 (42.9%) oncologists did. The St. Gallen Guidelines were used by 6 (85.7%) oncologists but only by 38 (47.5%) pathologists. The NCCN Guidelines were mostly used by 17 (89,5%) radiation oncologists, but only by 7 (8.8%) pathologists. The ASTRO Guidelines were used by 8 (42.1%) radiation oncologists but none (0%) of the radiologists. The TNM Classification (UICC) was used by almost all (*n* = 79, 98.8%) pathologists but only 4 (57.1%) oncologists. The AJCC Cancer Manual was mostly used by oncologists (*n* = 6, 85.7%) and only by 9 (10.2%) surgeons.

**Table 1:** Results of the “General Section” of the survey for the different medical specialties

	**Surgery** **(*N* = 88)**	**Pathology (*N* = 80)**	**Radiation oncology (*N* = 19)**	**Radiology (*N* = 8)**	**Oncology (*N* = 7)**	**Total (*N* = 202)**	***p* value**
**A3. Guidelines/classifications for guidance**							
S3							< 0.001
Yes	81 (92.0%)	54 (67.5%)	19 (100.0%)	6 (75.0%)	3 (42.9%)	163 (80.7%)	
No	7 (8.0%)	26 (32.5%)	0 (0.0%)	2 (25.0%)	4 (57.1%)	39 (19.3%)	
St. Gallen							0.003
Yes	66 (75.0%)	38 (47.5%)	11 (57.9%)	4 (50.0%)	6 (85.7%)	125 (61.9%)	
No	22 (25.0%)	42 (52.5%)	8 (42.1%)	4 (50.0%)	1 (14.3%)	77 (38.1%)	
ASBrS							0.455
Yes	2 (2.3%)	3 (3.8%)	2 (10.5%)	0 (0.0%)	0 (0.0%)	7 (3.5%)	
No	86 (97.7%)	77 (96.2%)	17 (89.5%)	8 (100.0%)	7 (100.0%)	195 (96.5%)	
NCCN							< 0.001
Yes	23 (26.1%)	7 (8.8%)	17 (89.5%)	1 (12.5%)	5 (71.4%)	53 (26.2%)	
No	65 (73.9%)	73 (91.2%)	2 (10.5%)	7 (87.5%)	2 (28.6%)	149 (73.8%)	
ISBrC							1.000
Yes	1 (1.1%)	1 (1.2%)	0 (0.0%)	0 (0.0%)	0 (0.0%)	2 (1.0%)	
No	87 (98.9%)	79 (98.8%)	19 (100.0%)	8 (100.0%)	7 (100.0%)	200 (99.0%)	
ASTRO							< 0.001
Yes	19 (21.6%)	5 (6.2%)	8 (42.1%)	0 (0.0%)	2 (28.6%)	34 (16.8%)	
No	69 (78.4%)	75 (93.8%)	11 (57.9%)	8 (100.0%)	5 (71.4%)	168 (83.2%)	
TNM (UICC)							< 0.001
Yes	75 (85.2%)	79 (98.8%)	16 (84.2%)	6 (75.0%)	4 (57.1%)	180 (89.1%)	
No	13 (14.8%)	1 (1.2%)	3 (15.8%)	2 (25.0%)	3 (42.9%)	22 (10.9%)	
AJCC							< 0.001
Yes	9 (10.2%)	21 (26.2%)	3 (15.8%)	1 (12.5%)	6 (85.7%)	40 (19.8%)	
No	79 (89.8%)	59 (73.8%)	16 (84.2%)	7 (87.5%)	1 (14.3%)	162 (80.2%)	
**A4. R Classification (TNM, UICC) provides information about**							
Residual tumour after therapy							0.719
Yes^a^	54 (62.1%)	50 (62.5%)	10 (52.6%)	3 (50.0%)	5 (83.3%)	122 (61.6%)	
No	33 (37.9%)	30 (37.5%)	9 (47.4%)	3 (50.0%)	1 (16.7%)	76 (38.4%)	
Prognosis							0.433
Yes^a^	35 (40.2%)	41 (51.9%)	8 (42.1%)	2 (33.3%)	4 (66.7%)	90 (45.7%)	
No	52 (59.8%)	38 (48.1%)	11 (57.9%)	4 (66.7%)	2 (33.3%)	107 (54.3%)	
Only resection margin of primary tumour							0.002
Yes	67 (77.0%)	39 (48.8%)	14 (73.7%)	5 (83.3%)	4 (66.7%)	129 (65.2%)	
No^a^	20 (23.0%)	41 (51.2%)	5 (26.3%)	1 (16.7%)	2 (33.3%)	69 (34.8%)	
Involvement of distant metastasis							< 0.001
Yes^a^	11 (12.6%)	40 (50.6%)	1 (5.3%)	1 (16.7%)	2 (33.3%)	55 (27.9%)	
No	76 (87.4%)	39 (49.4%)	18 (94.7%)	5 (83.3%)	4 (66.7%)	142 (72.1%)	
**A5. Familiarity with R category (TNM, UICC)**							
RX							0.054
Yes	79 (91.9%)	79 (98.8%)	18 (94.7%)	5 (83.3%)	5 (83.3%)	186 (94.4%)	
No	7 (8.1%)	1 (1.2%)	1 (5.3%)	1 (16.7%)	1 (16.7%)	11 (5.6%)	
R0							0.810
Yes	84 (96.6%)	79 (98.8%)	19 (100.0%)	6 (100.0%)	6 (100.0%)	194 (98.0%)	
No	3 (3.4%)	1 (1.2%)	0 (0.0%)	0 (0.0%)	0 (0.0%)	4 (2.0%)	
R0 > 1 mm							0.301
Yes	46 (52.9%)	47 (59.5%)	15 (78.9%)	3 (50.0%)	3 (50.0%)	114 (57.9%)	
No	41 (47.1%)	32 (40.5%)	4 (21.1%)	3 (50.0%)	3 (50.0%)	83 (42.1%)	
R0 ≤ 1 mm							0.172
Yes	43 (50.0%)	48 (60.8%)	15 (78.9%)	3 (50.0%)	3 (50.0%)	112 (57.1%)	
No	43 (50.0%)	31 (39.2%)	4 (21.1%)	3 (50.0%)	3 (50.0%)	84 (42.9%)	
R1							0.468
Yes	84 (96.6%)	77 (96.2%)	19 (100.0%)	5 (83.3%)	6 (100.0%)	191 (96.5%)	
No	3 (3.4%)	3 (3.8%)	0 (0.0%)	1 (16.7%)	0 (0.0%)	7 (3.5%)	
R2							< 0.001
Yes	61 (70.1%)	67 (84.8%)	18 (94.7%)	1 (16.7%)	5 (83.3%)	152 (77.2%)	
No	26 (29.9%)	12 (15.2%)	1 (5.3%)	5 (83.3%)	1 (16.7%)	45 (22.8%)	
R2a							0.568
Yes	11 (12.6%)	11 (13.9%)	3 (15.8%)	2 (33.3%)	1 (16.7%)	28 (14.2%)	
No	76 (87.4%)	68 (86.1%)	16 (84.2%)	4 (66.7%)	5 (83.3%)	169 (85.8%)	
R2b							0.519
Yes	11 (12.6%)	10 (12.7%)	3 (15.8%)	2 (33.3%)	1 (16.7%)	27 (13.7%)	
No	76 (87.4%)	69 (87.3%)	16 (84.2%)	4 (66.7%)	5 (83.3%)	170 (86.3%)	
R2c							0.945
Yes	5 (5.7%)	4 (5.1%)	0 (0.0%)	0 (0.0%)	0 (0.0%)	9 (4.6%)	
No	82 (94.3%)	75 (94.9%)	19 (100.0%)	6 (100.0%)	6 (100.0%)	188 (95.4%)	
**A6. Use of R category (TNM, UICC)**							
RX							0.267
Yes	62 (72.9%)	67 (83.8%)	17 (89.5%)	4 (66.7%)	5 (83.3%)	155 (79.1%)	
No	23 (27.1%)	13 (16.2%)	2 (10.5%)	2 (33.3%)	1 (16.7%)	41 (20.9%)	
R0							0.057
Yes	85 (100.0%)	76 (95.0%)	19 (100.0%)	5 (83.3%)	6 (100.0%)	191 (97.4%)	
No	0 (0.0%)	4 (5.0%)	0 (0.0%)	1 (16.7%)	0 (0.0%)	5 (2.6%)	
R0 > 1 mm							0.392
Yes	30 (36.1%)	24 (30.4%)	10 (52.6%)	2 (33.3%)	1 (16.7%)	67 (34.7%)	
No	53 (63.9%)	55 (69.6%)	9 (47.4%)	4 (66.7%)	5 (83.3%)	126 (65.3%)	
R0 ≤ 1 mm							0.389
Yes	31 (36.9%)	24 (30.4%)	10 (52.6%)	2 (33.3%)	1 (16.7%)	68 (35.1%)	
No	53 (63.1%)	55 (69.6%)	9 (47.4%)	4 (66.7%)	5 (83.3%)	126 (64.9%)	
R1							0.349
Yes	82 (97.6%)	77 (96.2%)	19 (100.0%)	5 (83.3%)	6 (100.0%)	189 (96.9%)	
No	2 (2.4%)	3 (3.8%)	0 (0.0%)	1 (16.7%)	0 (0.0%)	6 (3.1%)	
R2							0.001
Yes	48 (57.8%)	55 (69.6%)	18 (94.7%)	1 (16.7%)	5 (83.3%)	127 (65.8%)	
No	35 (42.2%)	24 (30.4%)	1 (5.3%)	5 (83.3%)	1 (16.7%)	66 (34.2%)	
R2a							0.064
Yes	6 (7.1%)	1 (1.3%)	1 (5.3%)	1 (16.7%)	1 (16.7%)	10 (5.2%)	
No	78 (92.9%)	78 (98.7%)	18 (94.7%)	5 (83.3%)	5 (83.3%)	184 (94.8%)	
R2b							0.058
Yes	6 (7.1%)	1 (1.3%)	1 (5.3%)	1 (16.7%)	1 (16.7%)	10 (5.2%)	
No	78 (92.9%)	78 (98.7%)	18 (94.7%)	5 (83.3%)	5 (83.3%)	184 (94.8%)	
R2c							0.449
Yes	3 (3.6%)	0 (0.0%)	0 (0.0%)	0 (0.0%)	0 (0.0%)	3 (1.6%)	
No	80 (96.4%)	79 (100.0%)	19 (100.0%)	6 (100.0%)	6 (100.0%)	190 (98.4%)	
**A7. Perception R categories (TNM, UICC) clearly defined**							0.311
Yes	60 (72.3%)	45 (57.7%)	12 (63.2%)	5 (83.3%)	3 (60.0%)	125 (65.4%)	
No	23 (27.7%)	33 (42.3%)	7 (36.8%)	1 (16.7%)	2 (40.0%)	66 (34.6%)	
**A8. Description of R category (TNM, UICC)**							0.005
Not satisfactory	21 (24.7%)	40 (50.0%)	4 (21.1%)	1 (16.7%)	2 (40.0%)	68 (34.9%)	
Satisfactory	64 (75.3%)	40 (50.0%)	15 (78.9%)	5 (83.3%)	3 (60.0%)	127 (65.1%)	
**A9. Who should carry out R Classification?**							0.394
Attending physician most familiar with the patient’s history^a^	0 (0%)	0 (0%)	0 (0%)	0 (0%)	0 (0%)	0 (0%)	
Together in the tumour board	38 (44.2%)	44 (55.0%)	5 (26.3%)	3 (50.0%)	2 (33.3%)	92 (46.7%)	
Surgeon	4 (4.7%)	2 (2.5%)	0 (0.0%)	0 (0.0%)	0 (0.0%)	6 (3.0%)	
Pathologist	44 (51.2%)	34 (42.5%)	14 (73.7%)	3 (50.0%)	4 (66.7%)	99 (50.3%)	

Missing answers are not shown explicitly but are the difference to the given total number. Percentages refer to all given answers

^a^Correct answer

Significant differences between medical specialties were also seen with question A4 regarding the general understanding of the R Classification, consisting of four different statements that each had to be agreed or disagreed with: Whereas there were no significant differences between the medical specialties for two of these statements (prognostic relevance and residual tumour after therapy), 67 (77.0%) surgeons but only 39 (48.8%) pathologists believe that the R Classification of the UICC provides information only about the resection margin of the primary tumour, a difference that was significant in the post hoc analyses. Furthermore, there were significant differences between surgeons and pathologists as well as between pathologists and radiation oncologists regarding whether the R Classification of the UICC has to include information about distant metastasis; whereas 40 (50.6%) pathologists correctly agreed with the statement, only 11 (12.6%) surgeons and 1 (5.3%) radiation oncologist did. In a “sum score”, four points could be scored for question A4. Only 1 (1.1%) surgeon, 20 (25.3%) pathologists, and 1 (16.7%) oncologist scored full points. The only statistically significant difference after Bonferroni correction was seen between pathologists (median 2, range 0–4) and surgeons (median 1, range 0–4).

Whereas most responding physicians (*n* = 125, 65.4%) perceived the R categories of the UICC as clearly defined, there were significant post hoc differences regarding satisfaction with practical use of the R categories between surgeons (*n* = 64, 75.3% satisfied) and pathologists (*n* = 40, 50.0% satisfied).

Although there were no significant differences regarding which medical specialty should carry out the R Classification, no-one (0%) gave the “correct” answer, namely the “attending physician most familiar with the patient’s history”.

### Section “General TNM Classification”

The results are summarized in Table 2. In this section, almost all questions had an objective “correct” answer. For question B6, the “correct” R category for a case after neoadjuvant therapy with a tumour-free scarred area of the primary tumour located at the resection margin, significant differences between groups for the original answer categories (*p* = 0.002) were observed as well as regarding correctly answered or not (*p* = 0.043). Whereas with the original categories, after Bonferroni correction there were significant differences between pathologists and surgeons as well as radiation oncologists, there were no significant post hoc differences regarding “correct”/”false”.

**Table 2:** Results of the “General TNM Section” of the survey for the different medical specialties

	**Surgery (*N* = 88)**	**Pathology (*N* = 80)**	**Radiation oncology (*N* = 19)**	**Radiology (*N* = 8)**	**Oncology (*N* = 7)**	**Total (*N* = 202)**	***p* value**
**B1. Besides margin further information for R0 needed**							0.166
Yes^a^	43 (58.1%)	53 (73.6%)	11 (57.9%)	2 (40.0%)	4 (80.0%)	113 (64.6%)	
No	31 (41.9%)	19 (26.4%)	8 (42.1%)	3 (60.0%)	1 (20.0%)	62 (35.4%)	
**B2. Breast resection specimen, tumour-free margin, positive removed sentinel, no axillary dissection**^**b**^							0.120
No R category	2 (2.7%)	10 (13.5%)	1 (5.3%)	0 (0.0%)	0 (0.0%)	13 (7.3%)	
R0^a^	68 (90.7%)	55 (74.3%)	18 (94.7%)	4 (80.0%)	5 (100.0%)	150 (84.3%)	
RX	5 (6.7%)	9 (12.2%)	0 (0.0%)	1 (20.0%)	0 (0.0%)	15 (8.4%)	
**B3. Resection specimen in several parts**^**b**^							0.138
No R category	21 (28.0%)	18 (24.0%)	4 (21.1%)	2 (40.0%)	1 (20.0%)	46 (25.7%)	
R0	19 (25.3%)	6 (8.0%)	5 (26.3%)	1 (20.0%)	1 (20.0%)	32 (17.9%)	
R1	0 (0.0%)	1 (1.3%)	0 (0.0%)	0 (0.0%)	0 (0.0%)	1 (0.6%)	
RX^a^	35 (46.7%)	50 (66.7%)	10 (52.6%)	2 (40.0%)	3 (60.0%)	100 (55.9%)	
**B4. Resection specimen with torn surface, poorly assessable margins**^**b**^							0.939
No R category	14 (18.9%)	15 (20.3%)	3 (15.8%)	1 (20.0%)	2 (40.0%)	35 (19.8%)	
R0	1 (1.4%)	1 (1.4%)	0 (0.0%)	0 (0.0%)	0 (0.0%)	2 (1.1%)	
R1	0 (0.0%)	1 (1.4%)	0 (0.0%)	0 (0.0%)	0 (0.0%)	1 (0.6%)	
RX^a^	59 (79.7%)	57 (77.0%)	16 (84.2%)	4 (80.0%)	3 (60.0%)	139 (78.5%)	
**B5. No tumour detectable in resection specimen after neoadjuvant therapy**^**b**^							0.066
No R category	26 (35.1%)	15 (20.0%)	8 (42.1%)	0 (0.0%)	2 (40.0%)	51 (28.7%)	
R0^a^	45 (60.8%)	59 (78.7%)	11 (57.9%)	4 (80.0%)	3 (60.0%)	122 (68.5%)	
RX	3 (4.1%)	1 (1.3%)	0 (0.0%)	1 (20.0%)	0 (0.0%)	5 (2.8%)	
**B6. Resection specimen after neoadjuvant therapy scarred, tumour-free area at margin**^**b**^							0.002
No R category	16 (21.3%)	13 (17.3%)	8 (42.1%)	0 (0.0%)	2 (40.0%)	39 (21.8%)	
R0^a^	42 (56.0%)	51 (68.0%)	6 (31.6%)	2 (40.0%)	3 (60.0%)	104 (58.1%)	
R1	10 (13.3%)	0 (0.0%)	2 (10.5%)	1 (20.0%)	0 (0.0%)	13 (7.3%)	
R2	1 (1.3%)	0 (0.0%)	0 (0.0%)	0 (0.0%)	0 (0.0%)	1 (0.6%)	
RX	6 (8.0%)	11 (14.7%)	3 (15.8%)	2 (40.0%)	0 (0.0%)	22 (12.3%)	
**B7. Resection specimen, neoadjuvant therapy, residual tumour cell nests distributed 5 mm and more, at margin scar tissue, nearest residual tumour a distance of 2 mm to margin**^**b**^							0.103
No R category	5 (6.7%)	12 (15.8%)	3 (15.8%)	0 (0.0%)	1 (20.0%)	21 (11.7%)	
R0^a^	43 (57.3%)	38 (50.0%)	8 (42.1%)	2 (40.0%)	3 (60.0%)	94 (52.2%)	
R1	11 (14.7%)	5 (6.6%)	4 (21.1%)	1 (20.0%)	1 (20.0%)	22 (12.2%)	
R2	3 (4.0%)	0 (0.0%)	0 (0.0%)	1 (20.0%)	0 (0.0%)	4 (2.2%)	
RX	13 (17.3%)	21 (27.6%)	4 (21.1%)	1 (20.0%)	0 (0.0%)	39 (21.7%)	

Missing answers are not shown explicitly but are the difference to the given total number. Percentages refer to all given answers

^a^Correct answer

^b^Possible answers were “no R category”, “R0”, “R1”, “R2”, and “RX”

### Section “Specific Questions Regarding Breast Cancer Diagnosis”

The results are summarized in Table 3. There were six possible answers, with multiple answers allowed, to the question what category R1 stands for in breast surgery (question C1). Viewed individually, there was a significant difference for one of the options, namely that R1 in breast surgery means invasive carcinoma at inked margin (*p* < 0.001). Whereas all pathologists correctly answered in agreement, 62 (83.8%) surgeons and 3 (60.0%) radiologists did; both differences were significant after Bonferroni correction.

**Table 3:** Results of the “Specific Questions Regarding Breast Cancer Diagnosis Section” of the survey for the different medical specialties

	**Surgery (*N* = 88)**	**Pathology (*N* = 80)**	**Radiation oncology (*N* = 19)**	**Radiology (*N* = 8)**	**Oncology (*N* = 7)**	**Total (*N* = 202)**	***p* value**
**C1. R1 in breast surgery: (multiple answers possible)**							
Invasive carcinoma at inked margin							< 0.001
Yes	62 (83.8%)	73 (100.0%)	14 (87.5%)	3 (60.0%)	4 (80.0%)	156 (90.2%)	
No	12 (16.2%)	0 (0.0%)	2 (12.5%)	2 (40.0%)	1 (20.0%)	17 (9.8%)	
Invasive carcinoma fractions of a millimetre to margin							0.214
Yes	12 (16.2%)	7 (9.6%)	3 (18.8%)	2 (40.0%)	0 (0.0%)	24 (13.9%)	
No	62 (83.8%)	66 (90.4%)	13 (81.2%)	3 (60.0%)	5 (100.0%)	149 (86.1%)	
Invasive carcinoma less than 2 mm to margin							0.110
Yes	6 (8.1%)	0 (0.0%)	0 (0.0%)	0 (0.0%)	0 (0.0%)	6 (3.5%)	
No	68 (91.9%)	73 (100.0%)	16 (100.0%)	5 (100.0%)	5 (100.0%)	167 (96.5%)	
Invasive carcinoma more than 2 mm to the resection margin, but DCIS at margin							0.224
Yes	22 (29.7%)	31 (42.5%)	5 (31.2%)	1 (20.0%)	0 (0.0%)	59 (34.1%)	
No	52 (70.3%)	42 (57.5%)	11 (68.8%)	4 (80.0%)	5 (100.0%)	114 (65.9%)	
DCIS at inked margin							0.687
Yes	51 (68.9%)	56 (76.7%)	12 (75.0%)	3 (60.0%)	3 (60.0%)	125 (72.3%)	
No	23 (31.1%)	17 (23.3%)	4 (25.0%)	2 (40.0%)	2 (40.0%)	48 (27.7%)	
DCIS (no invasive tumour) less than 2 mm to margin							0.070
Yes	29 (39.2%)	14 (19.2%)	3 (18.8%)	1 (20.0%)	1 (20.0%)	48 (27.7%)	
No	45 (60.8%)	59 (80.8%)	13 (81.2%)	4 (80.0%)	4 (80.0%)	125 (72.3%)	
**C2. Neoadjuvant treated breast carciNoma, disseminated growth, single tumour cells do Not reach margin**^**b**^							0.113
No R category	10 (13.7%)	12 (16.4%)	2 (12.5%)	0 (0.0%)	1 (25.0%)	25 (14.6%)	
R0^a^	51 (69.9%)	38 (52.1%)	12 (75.0%)	3 (60.0%)	3 (75.0%)	107 (62.6%)	
R1	3 (4.1%)	3 (4.1%)	0 (0.0%)	2 (40.0%)	0 (0.0%)	8 (4.7%)	
RX	9 (12.3%)	20 (27.4%)	2 (12.5%)	0 (0.0%)	0 (0.0%)	31 (18.1%)	
**C3. Operated breast carcinoma with tumour-free margin, not operated liver metastasis**^**b**^							< 0.001
No R category	4 (5.5%)	9 (12.5%)	0 (0.0%)	1 (20.0%)	1 (20.0%)	15 (8.8%)	
R0	64 (87.7%)	36 (50.0%)	15 (93.8%)	4 (80.0%)	2 (40.0%)	121 (70.8%)	
R1	1 (1.4%)	1 (1.4%)	0 (0.0%)	0 (0.0%)	0 (0.0%)	2 (1.2%)	
R2^**a**^	3 (4.1%)	25 (34.7%)	1 (6.2%)	0 (0.0%)	2 (40.0%)	31 (18.1%)	
RX	1 (1.4%)	1 (1.4%)	0 (0.0%)	0 (0.0%)	0 (0.0%)	2 (1.2%)	

Missing answers are not shown explicitly but are the difference to the given total number. Percentages refer to all given answers

^a^Correct answer

^b^Possible answers were “no R category”, “R0”, “R1”, “R2”, and “RX”

Question C3 for an R category in an operated breast carcinoma specimen with a widely tumour-free resection margin and histologically verified but not operated liver metastasis also showed significant group differences (*p* < 0.001 both for original answer categories as well as “correct”/”false”). Only 18.1% physicians answered the question correctly, with a significant post hoc difference between surgeons (*n* = 3, 4.1%) and pathologists (*n* = 25, 34.7%).

**Fig. 1:**
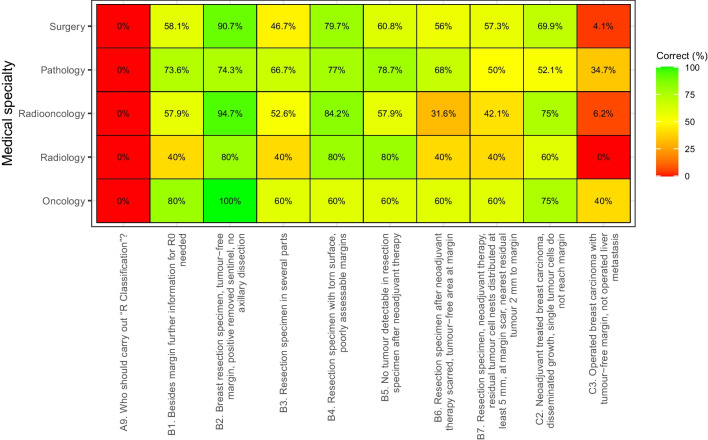
Heatmap of the questions with an objective “correct” answer according to guidelines; shown are percentages of correct answers per specialty. Correct answers to the questions: A9: attending physician most familiar with the patient’s history, B1: yes, B2: R0, B3: RX, B4: RX, B5: R0, B6: R0, B7: R0, C2: R0, and C3: R2

The distribution of the percentages of “correct” answers by specialty is presented in Fig. 1 as a heatmap.

The results regarding professional experience are not treated here since experience and specialty were not independent.

## Discussion

In addition to the histopathological diagnosis including the “TNM Classification of Malignant Tumours”, physicians expect a final R Classification from the pathologist (see Table 4), although the R Classification is not included in the recommendations of the International Collaboration on Cancer reporting (ICCR) dataset concerning breast cancer [29–31]. But nevertheless, based on our survey, as 89.1% physicians use the TNM system of the UICC and based on the national TNM committees, operating throughout the world [4], the problem with the R classification in breast carcinomas likely exists worldwide, since the R Classification is a required and integral part of the TNM Classification.

**Table 4:** Definitions of the R categories of the UICC

TNM Classification of Malignant Tumours, 8th Edition, UICC(3)	TNM Supplement, 5th Edition, UICC(4)	Definitions
RX	RX	“Presence of residual tumour cannot be assessed”
R0		“No residual tumour”
	R0(un)	“Uncertain resection”, “no macroscopic or microscopic evidence of residual disease” but “nodal assessment has been based on less than the number of lymph nodes/stations ordinarily included in a lymphadenectomy specimen” or “the highest mediastinal node removed/sampled is positive (for lung cancers)”
	R0 > 1 mm	“No residual tumour, minimal distance between tumour and resection margin > 1 mm”
	R0 ≤ 1 mm	“No residual tumour, minimal distance between tumour and resection margin ≤ 1 mm”
R1		“Microscopic residual tumour”
	R1‐dir	“Microscopic residual tumour, tumour directly at the resection margin (tumour transected)”
“R2”		“Macroscopic residual tumour”
	“R2a”	“Local macroscopic residual tumour”
	“R2b”	“Distant macroscopic residual tumour”
	“R2c”	“Macroscopic residual tumour in both sites”

The complex issue of resection margin in breast carcinomas [16–24] and the R Classification [3, 4] often lead to discussions amongst the attending physicians. One major finding in our survey was that even by experienced physicians the R Classification and the resection margin status are often mixed up. It is hardly surprising that there is so much confusion, since even in the German AGO S3 Guideline the resection margin is equated with the R Classification [16], in contrast to the UICC definition [4].

The standard for an adequate margin in the common guidelines for invasive breast cancer with or without DCIS is “no ink on tumour”; for a pure DCIS treated by BCS the optimal margin width should be at least 2 mm. These recommendations apply only to whole breast irradiation [16–21, 23, 24]. These suggestions reduce the risk of local recurrence, but even if the resection margins are enlarged there is no statistically significant decrease for local recurrence [20, 32]. Neither in invasive carcinoma nor in DCIS does a negative margin guarantee the absence of residual tumour in the breast [20, 21]. Subclinical foci of tumour tissue in the surrounding breast tissue may be present at large distances from the primary tumour [33] and in DCIS the involvement of the segment may be multifocal with “gaps” of uninvolved tissue between the DCIS foci [34]. Additionally, the sequential embedding results in the examination of only < 1% of specimen margin [35], and the inked tumour-free margin may not show the true state of the margin 3-dimensionally. Therefore, an adequate margin may actually be positive if deeper sections are cut from the same tissue block [20, 21]. Nevertheless, a negative margin indicates that the residual tumour burden is low enough to be controlled with radiotherapy [15].

In addition, it is hardly known that distant metastases are also included in the R Classification. Even though a similar question is used as an example in the TNM Supplement [4], only 18.1% of physicians answered question C3 correctly. This result is striking but not surprising since the TNM Classification of the UICC also contains contradictions and unclear statements. The supplement clearly states that not only the locoregional residual tumour is included but also the distant residual tumour in form of remaining metastases. Moreover, metastases should be included because of their prognostic relevance [4]. However, in the TNM Classification a comment explains that the use should be further specified because some use “R” to apply only to the primary tumour with its local extent, whereas others include distant metastases [3].

Another alarming finding of our survey was regarding the creation of the R Classification. Wittekind et al. propose that “the R classification must be performed by a designated individual who has access to the complete data [6]”. This person could be a surgeon, medical oncologist, radiation oncologist, tumour registrar, or pathologist. Without complete clinical data provided by the surgeon to the pathologist, the pathologist should only state the resection margin status [6].

Finally, one problem still remains regarding positive sentinel nodes without an axillary dissection. Wittekind proposes that a specimen with a lymphadenectomy should be classified as R0 unless the positive node is transected. But how should physicians proceed if only a sentinel lymph node is present? We suggest the use of R0(un) in these cases although this category seems to be used mostly for lung cancer surgery [4, 36, 37] if there are too few nodes/stations in a lymphadenectomy specimen or if the highest mediastinal node is positive for cancer [4].

### Limitations

Due to the vastly different sample sizes for the various specialties, most significant post hoc differences were found between the largest groups only, even if there were larger differences between smaller groups. Finally, our survey was mostly worded asking for opinions, which could lead to a mixture of participants not knowing the guidelines and definitions or simply being of another opinion. Therefore, we have made the analysis of “correct” and “false” a secondary analysis and use quotation marks for these terms.

## Conclusion

For the first time, we were able to show that there are widespread significant differences in the interpretation of the R Classification between different medical disciplines.

In review of the most recent literature and guidelines, we would highly recommend that pathologists include an assessment of the resection margin status in their final histopathology report without a further statement to an overall R Classification. More precisely, we suggest the use of a Resection margin (Rm) Classification, which equals in general the R categories of the UICC but is limited to the pathological viewpoint, namely resection margin evaluation only. However, in contrast to the UICC building on this Rm Classification, the multidisciplinary team, which is aware of all clinical data, imaging results, histology, cytology and molecular reports as well as the history of the patient should carry out a final R Classification.

Unclear cases should be reported to the TNM Help desk [38] and discussed multidisciplinary afterwards because it is high time that all medical disciplines should be educated about the actual meaning and correct application of the R Classification. This is to assist better multidisciplinary cooperation and thus to ensure better patient care.

## Supplementary Information

Below is the link to the electronic supplementary material.

Supplementary file1 (DOCX 27 KB)

## Data Availability

The data that support the findings of this study are available from the corresponding author upon reasonable request.

## References

[1] WHO Classification of Tumours Editoral Board (2019) Breast tumours. Lyon: Int Agency Res Cancer

[2] Veronesi U, Cascinelli N, Mariani L, Greco M, Saccozzi R, Luini A et al (2002) Twenty-year follow-up of a randomized study comparing breast-conserving surgery with radical mastectomy for early breast cancer. N Engl J Med 347(16):1227–1232 12393819 10.1056/NEJMoa020989

[3] UICC (Union for International Cancer Control) (2017) TNM Classification of Malignant Tumours, 8th edn, Brierley JD, Gospadarowicz MK, Wittekind C (eds). Oxford: Wiley Blackwell

[4] UICC (Union for International Cancer Control) (2019) TNM Supplement. A commentary on uniform use, 5th edn, Wittekind Ch, Brierley JD, Lee A.W.M. Eycken, E. (eds). Hoboken: Wiley Blackwell

[5] UICC (Union for International Cancer Control) (1993) TNM Supplement 1993. A Commentary on Uniform Use, Hermanek P, Henson DE, Hutter RVP, Sohin LH (eds). Berlin, Heidelberg, New York: Springer

[6] Wittekind C, Compton CC, Greene FL, Sobin LH (2002) TNM residual tumor classification revisited. Cancer 94(9):2511–2516 12015777 10.1002/cncr.10492

[7] Wittekind C (2007) Problems with residual tumor classification, particularly R1. Chirurg 78(9):785–791 17676285 10.1007/s00104-007-1378-5

[8] Hermanek P, Wittekind C (1994) Residual tumor (R) classification and prognosis. Semin Surg Oncol 10(1):12–20 8115781 10.1002/ssu.2980100105

[9] Hermanek P, Wittekind C (1994) The pathologist and the residual tumor (R) classification. Pathol Res Pract 190(2):115–123 8058567 10.1016/S0344-0338(11)80700-4

[10] Wittekind C, Compton C, Quirke P, Nagtegaal I, Merkel S, Hermanek P et al (2009) A uniform residual tumor (R) classification: integration of the R classification and the circumferential margin status. Cancer 115(15):3483–3488 19536900 10.1002/cncr.24320

[11] Bundred JR, Michael S, Stuart B, Cutress RI, Beckmann K, Holleczek B et al (2022) Margin status and survival outcomes after breast cancer conservation surgery: prospectively registered systematic review and meta-analysis. BMJ 378:e070346 36130770 10.1136/bmj-2022-070346 PMC9490551

[12] Houssami N, Macaskill P, Marinovich ML, Morrow M (2014) The association of surgical margins and local recurrence in women with early-stage invasive breast cancer treated with breast-conserving therapy: a meta-analysis. Ann Surg Oncol 21(3):717–730 24473640 10.1245/s10434-014-3480-5 PMC5705035

[13] Brouwer de Koning SG, VranckenPeeters M, Jozwiak K, Bhairosing PA, Ruers TJM (2018) Tumor resection margin definitions in breast-conserving surgery: systematic review and meta-analysis of the current literature. Clin Breast Cancer 18(4):e595–e600 29731404 10.1016/j.clbc.2018.04.004

[14] Haloua MH, Volders JH, Krekel NM, Barbe E, Sietses C, Jozwiak K et al (2016) A nationwide pathology study on surgical margins and excision volumes after breast-conserving surgery: There is still much to be gained. Breast 25:14–21 26801411 10.1016/j.breast.2015.11.003

[15] Pilewskie M, Morrow M (2018) Margins in breast cancer: how much is enough? Cancer 124(7):1335–1341 29338088 10.1002/cncr.31221 PMC5894883

[16] Evidence-based guideline for the early detection, diagnosis, treatment and follow-up of breast cancer: German Guideline Program in Oncology (German Cancer Society, German Cancer Aid, AWMF); 2021 [Available from: https://www.leitlinienprogramm-onkologie.de/leitlinien/mammakarzinom/

[17] NCCN Clinical Practice Guidelines in Oncology (NCCN Guidelines®) Breast Cancer: National Comprehensive Cancer Network; 2022 [Available from: https://www.nccn.org/professionals/physician_gls/pdf/breast.pdf

[18] Consensus guideline on breast cancer lumpectomy margins: the American Society of Breast Surgeons; 2017 [Available from: https://www.breastsurgeons.org/docs/statements/Consensus-Guideline-on-Breast-Cancer-Lumpectomy-Margins.pdf

[19] Burstein HJ, Curigliano G, Loibl S, Dubsky P, Gnant M, Poortmans P et al (2019) Estimating the benefits of therapy for early-stage breast cancer: the St. Gallen International Consensus Guidelines for the primary therapy of early breast cancer 2019. Ann Oncol 30(10):1541–57 31373601 10.1093/annonc/mdz235

[20] Moran MS, Schnitt SJ, Giuliano AE, Harris JR, Khan SA, Horton J et al (2014) Society of Surgical Oncology-American Society for Radiation Oncology consensus guideline on margins for breast-conserving surgery with whole-breast irradiation in stages I and II invasive breast cancer. Int J Radiat Oncol Biol Phys 88(3):553–564 24521674 10.1016/j.ijrobp.2013.11.012 PMC4790083

[21] Morrow M, Van Zee KJ, Solin LJ, Houssami N, Chavez-MacGregor M, Harris JR et al (2016) Society of Surgical Oncology-American Society for Radiation Oncology-American Society of Clinical Oncology Consensus Guideline on Margins for breast-conserving surgery with whole-breast irradiation in ductal carcinoma in situ. Pract Radiat Oncol 6(5):287–295 27538810 10.1016/j.prro.2016.06.011 PMC5070537

[22] Maloney BW, McClatchy DM, Pogue BW, Paulsen KD, Wells WA, Barth RJ (2018) Review of methods for intraoperative margin detection for breast conserving surgery. J Biomed Opt 23(10):1–19 30369108 10.1117/1.JBO.23.10.100901 PMC6210801

[23] Buchholz TA, Somerfield MR, Griggs JJ, El-Eid S, Hammond ME, Lyman GH et al (2014) Margins for breast-conserving surgery with whole-breast irradiation in stage I and II invasive breast cancer: American Society of Clinical Oncology endorsement of the Society of Surgical Oncology/American Society for Radiation Oncology consensus guideline. J Clin Oncol 32(14):1502–1506 24711553 10.1200/JCO.2014.55.1572

[24] Tremelling A, Aft RL, Cyr AE, Gillanders WE, Glover-Collins K, Herrmann V et al (2022) Impact of consensus guidelines for breast-conserving surgery in patients with ductal carcinoma in situ. Cancer Rep (Hoboken) 5(5):e1502 34245135 10.1002/cnr2.1502 PMC9124516

[25] Statistik Austria; Accessed January 3, 2023[Available from: https://www.statistik.at/statistiken/bevoelkerung-und-soziales/bevoelkerung/bevoelkerungsstand/bevoelkerung-zu-jahres-/-quartalsanfang

[26] Statistische Ämter des Bundes und der Länder; Accessed January 3, 2023[Available from: https://www.statistikportal.de/de/bevoelkerung/flaeche-und-bevoelkerung

[27] Fischer P, Differences in the interpretation of the R classification -with a special focus on breast cancer, Master’s Thesis approved by Med Uni Graz, 2023

[28] LimeSurvey GmbH; 2021 [Available from: https://www.limesurvey.org/de/

[29] Fox S, Chen CJ, Chua B, Collins LC, Foschini MP, Mann B, Millar EKA, Pinder S, Rakha E, Shaaban AM, Tan BY, Tse GM, Watson PH, Tan PH (2021) Ductal carcinoma in situ, variants of lobular carcinoma in situ and low grade lesions histopathology reporting guide. International Collaboration on Cancer Reporting; Sydney, Australia 10.1111/his.14725 35869801

[30] Ellis I, Allison KH, Dang C, Gobbi H, Kulka J, Lakhani SR, Moriya T, Quinn CM, Sapino A, Schnitt S, Sibbering DM, Slodkowska E, Yang W, Tan PH (2022) Invasive carcinoma of the breast histopathology reporting guide, 2nd edition. International Collaboration on Cancer Reporting; Sydney, Australia

[31] Bossuyt V, Provenzano E, Symmans WF, Allison KH, Dang C, Gobbi H, Kulka J, Lakhani SR, Moriya T, Quinn CM, Sapino A, Schnitt S, Sibbering DM, Slodkowska E, Yang W, Tan PH, Ellis I (2022) Invasive carcinoma of the breast in the setting of neoadjuvant therapy histopathology reporting guide, 1st edition. International Collaboration on Cancer Reporting; Sydney, Australia

[32] Marinovich ML, Azizi L, Petra Macaskill P, Les Irwig L, Monica Morrow M, Solin LJ, Houssami N (2016) The association of surgical margins and local recurrence in women with ductal carcinoma in situ treated with breast-conserving therapy: a meta-analysis. Ann Surg Oncol 23(12):3811–3821 27527715 10.1245/s10434-016-5446-2 PMC5160992

[33] Holland R, Veling SH, Mravunac M, Hendriks JH (1985) Histologic multifocality of Tis, T1-2 breast carcinomas. Implications for clinical trials of breast-conserving surgery. Cancer 56(5):979–90 2990668 10.1002/1097-0142(19850901)56:5<979::aid-cncr2820560502>3.0.co;2-n

[34] Faverly DR, Burgers L, Bult P, Holland R (1994) Three dimensional imaging of mammary ductal carcinoma in situ: clinical implications. Semin Diagn Pathol 11(3):193–198 7831530

[35] Carter D (1986) Margins of “lumpectomy” for breast cancer. Hum Pathol 17(4):330–332 3957334 10.1016/s0046-8177(86)80455-5

[36] Rami-Porta R, Wittekind C, Goldstraw P, International Association for the Study of Lung Cancer Staging C (2005) Complete resection in lung cancer surgery: proposed definition. Lung Cancer 49(1):25–33 15949587 10.1016/j.lungcan.2005.01.001

[37] Gagliasso M, Migliaretti G, Ardissone F (2017) Assessing the prognostic impact of the International Association for the Study of Lung Cancer proposed definitions of complete, uncertain, and incomplete resection in non-small cell lung cancer surgery. Lung Cancer 111:124–130 28838382 10.1016/j.lungcan.2017.07.013

[38] UICC (Union for International Cancer Control) TNM Help desk; Accessed December 20, 2022[Available from: https://www.uicc.org/tnm-help-desk

