# Supplementary material for: The misunderstanding of the R Classification—a survey amongst medical specialties treating breast cancer
Source: Virchows Arch. 2024 Jul 22;485(3):479–90. doi: 10.1007/s00428-024-03876-8 (PMC11415394; doi:10.1007/s00428-024-03876-8)
Supplement: Supplementary file 1 — Supplementary file1 (DOCX 27 KB) [file 428_2024_3876_MOESM1_ESM.docx]

# S1

# Questionnaire

A1. In which medical specialty do you work?

□ Surgery/ Gynaecology

□ Radiation Oncology/ Therapy

□ Pathology

□ Oncology

□ Radiology

A2. How many years of professional experience do you have in your specialty (including residency)?

□ 0-5 years

□ 6-10 years

□ 11-15 years

□ ≥16 years

A3. Which of these Guidelines/ Classifications do you follow in your clinical work? (Multiple answers possible)

□ Evidence-based Guideline for the Early Detection, Diagnosis, Treatment and Follow-up of Breast Cancer – S3

□ St. Gallen International Consensus Guidelines for the primary therapy of early breast cancer

□ Consensus & Performance and Practice Guidelines of the American Society of Breast Surgeons – ASBrS

□ Clinical Practice Guidelines in Oncology for Breast Cancer of the National Comprehensive Cancer Network – NCCN

□ International Guideline for Management of Breast Cancer of the International Society of Breast Cancer – ISBrC

□ SSO/ASTRO/ASCO Consensus Guideline for DCIS and the SSO/ASTRO Consensus Guideline for Invasive Breast Cancer of the American Society for □ Radiation Oncology – ASTRO

□ TNM Classification of Malignant Tumours of the UICC

□ AJCC Cancer Staging Manual

A4. In your opinion, the R Classification of the UICC provides information about:

yes no

the residual tumour after therapy □ □

the prognosis □ □

only the resection margin of the primary tumour □ □

the involvement of distant metastasis □ □

A5. Are you familiar with the following R categories of the “TNM Classification of Malignant Tumours” of the UICC with the respective exact definition?

yes no

RX □ □

R0 □ □

R0>1mm □ □

R0≤1mm □ □

R1 □ □

R2 □ □

R2a □ □

R2b □ □

R2c □ □

A6. Which of the following R categories of the “TNM Classification of Malignant Tumours” of the UICC do you use in your daily clinical routine?

yes no

RX □ □

R0 □ □

R0>1mm □ □

R0≤1mm □ □

R1 □ □

R2 □ □

R2a □ □

R2b □ □

R2c □ □

A7. Do you consider the description of the R categories of the “TNM Classification of Malignant Tumours” of the UICC as clearly defined?

□ yes

□ no

A8. Do you consider the description of the R categories of the “TNM Classification of Malignant Tumours” of the UICC in practice as

□ satisfactory

□ not satisfactory

A9. Who do you think should carry out the R Classification?

□ Pathologist

□ Surgeon (surgeon/ gynaecologist)

□ Oncologist

□ The attending physician who is most familiar with the patient's medical history

□ Together interdisciplinary in the tumour board

B1. Besides the assessment of the resection margin, is there any additional information required for an R0 category?

□ yes

□ no

B2. Which R category would you assign, for a breast resection specimen with an invasive breast carcinoma with a tumour-free resection margin and a positive completely removed sentinel lymph node, but without an axillary dissection?

□ RX

□ R0

□ R1

□ R2

□ no R category can be assigned

B3. In your opinion, which R category is assigned if the resection specimen is sent in several parts?

□ RX

□ R0

□ R1

□ R2

□ no R category can be assigned

B4. In your opinion, which R category is assigned if the surgical specimen shows a multiple torn surface with partially poorly assessable resection margins?

□ RX

□ R0

□ R1

□ R2

□ no R category can be assigned

B5. In your opinion, which R category can be assigned if no tumour is detectable in a surgical specimen after neoadjuvant therapy?

□ RX

□ R0

□ R1

□ R2

□ no R category can be assigned

B6. In your opinion, which R category can be assigned if the scarred and now tumour-free area of the primary tumour after neoadjuvant therapy is located at the resection margin in a surgical specimen?

□ RX

□ R0

□ R1

□ R2

□ no R category can be assigned

B7. In your opinion, which R category can be assigned if in a surgical specimen after neoadjuvant therapy, there is a largely scarred area of the primary tumour. Residual tumour cell nests are distributed at a distance of 5 mm and more, the scarred tumour area is directly adjacent to the resection margin and the minimum distance to the margin of the nearest residual tumour cell nest is 2mm?

□ RX

□ R0

□ R1

□ R2

□ no R category can be assigned

C1. R1 in breast surgery means (multiple answers possible):

□ invasive carcinoma at inked margin

□ invasive carcinoma fractions of a millimetre to the resection margin

□ invasive carcinoma less than 2 mm to the resection margin

□ invasive carcinoma more than 2 mm to the resection margin, but DCIS at resection margin

□ DCIS at inked margin (without invasive tumour)

□ DCIS (without invasive tumour) less than 2 mm to the resection margin

C2. Which R category would you assign for a neoadjuvant treated breast carcinoma with disseminated growth pattern in the entire resection specimen (the single tumour cells do not reach the inked margin)?

□ RX

□ R0

□ R1

□ R2

□ no R category can be assigned

C3. Which R category would you assign for an operated breast carcinoma with a wide tumour-free resection margin and a clinically discovered (and histologically verified), but not operated liver metastasis?

□ RX

□ R0

□ R1

□ R2

□ no R category can be assigned
